# Do the elderly and those with comorbid chronic physical conditions have improved access to outpatient psychotherapy post structural reforms in Germany? Results of the ES-RiP study

**DOI:** 10.3389/fpsyt.2024.1349603

**Published:** 2024-04-29

**Authors:** Johanna Jedamzik, Hanna Kampling, Andrea Christoffer, Carsten Szardenings, Gereon Heuft, Hans-Christoph Friederich, Johannes Kruse

**Affiliations:** ^1^ Department of Psychosomatic Medicine and Psychotherapy, University Hospital Muenster, Muenster, Germany; ^2^ Department of Psychosomatic Medicine and Psychotherapy, Justus Liebig University Giessen, Giessen, Germany; ^3^ Institute of Biostatistics and Clinical Research, University of Muenster, Muenster, Germany; ^4^ Department of General Internal Medicine and Psychosomatics, University Hospital Heidelberg, Heidelberg, Germany; ^5^ Department for Psychosomatic Medicine and Psychotherapy, Medical Center of the Philipps University Marburg, Marburg, Germany

**Keywords:** comorbidity of mental and chronic physical disorders, health service, accessebility of psychotherapy, psychotherapy in the elderly, structural psychotherapy reform

## Abstract

**Background:**

In 2017, a reform of the German outpatient psychotherapy guideline was carried out, aiming to reduce waiting times and facilitate low-threshold access. This study analyzes the extent to which the implementation of the two new service elements ‘psychotherapeutic consultation times’ and ‘acute short-term psychotherapeutic interventions’ improved psychotherapeutic care for patients with mental disorders and chronic physical conditions (cMPs), for patients with mental disorders without chronic physical conditions (MnoP), and elderly patients.

**Methods:**

In a quantitative secondary analysis, we analyzed health insurance data of patients with psychotherapy billing codes obtained from the National Association of Statutory Health Insurance Physicians (KBV) for the years 2015-2019, evaluating descriptive statistical parameters for specific patient groups and care services.

**Results:**

Between 2015 and 2019, the number of mentally ill receiving psychotherapy at least once in the corresponding year increased by 30.7%. Among these, the proportion of cMPs-patients increased from 26.8% to 28.2% (+1.4%), while that of MnoP-patients decreased from 68.3% to 66.4% (-1.9%). The number of elderly people receiving treatment also increased.

**Conclusion:**

Since increases and decreases in the percentage shares occur evenly over the years investigated, it is questionable whether the reform in 2017 has had a direct influence on these changes.

**Study registration:**

ID DRKS00020344, URL: https://www.bfarm.de/DE/Das-BfArM/Aufgaben/Deutsches-Register-Klinischer-Studien/_node.html.

## 1 Introduction

The burden of mental disorders worldwide is high. In 2019, approximately 1 in every 8 people around the world suffered from mental disorders (1). In Germany, almost one third of the general population is affected by mental disorders every year (2). Simultaneously, large population surveys show a high prevalence of comorbidity between mental and physical disorders (3). In Europe, the prevalence of comorbidity between mental disorders and chronic physical conditions is increasing (4). Patients with chronic physical conditions are more likely to develop mental disorders than people without (5, 6). On the other hand, patients with mental disorders show a higher risk for somatic comorbidities and for a deterioration of physical conditions such as diabetes and cardiovascular diseases (7). The Lancet Commission on global mental health and sustainable development lists the focus on “comorbidity and multimorbidity across mental and physical long-term conditions” as pioneering aspect of global health care (8). Patients with a comorbidity of mental disorders and chronic physical conditions do have a significant lower quality of life, a prolonged length-of-stay in hospitals (9) as well as a significantly increased morbidity and mortality compared to patients without comorbid mental and physical health issues (3). Older people in particular represent a risk group in this regard, since chronic physical disorders are increasingly common with age. Therefore, there is an increased susceptibility to the negative effects of the mutual interaction of mental and chronic physical disorders. Comorbidity of mental disorders and chronic physical conditions is not only associated with significant suffering but also with higher health care costs, especially in multimorbid elderly patients (10).

However, irrespective of the increased need to address this comorbidity, patients with a comorbidity of mental disorders and chronic physical conditions (cMPs) – and especially elderly patients with cMPs – are an undersupplied group in outpatient psychotherapy (11).

With the aim to improve overall outpatient psychotherapeutic care and specifically the care of undersupplied groups, a nationwide reform of the psychotherapy guideline took place in Germany in 2017.

In Germany, costs for psychotherapeutic treatments are covered by private and statutory health insurance (SHI) and assumptions of costs for psychotherapeutic treatments are regulated by the psychotherapy guideline. Access to health insurance is possible for the whole population, independent of income. The reform of the psychotherapy guideline carried out in 2017 introduced two new structural elements namely, ‘psychotherapeutic consultation times’ and ‘acute short-term psychotherapeutic interventions’ (12). These two new billing codes supplement the previously existing item of the ‘probatory phase’ comprising up to six outpatient diagnostic probatory sessions including biographic anamnesis. These were not considered as psychotherapy sessions as far as formal cost structures covered by the psychotherapy guideline are concerned. Prior to this reform, the psychotherapist was obliged to submit an application for therapy on behalf of his patient based on the findings of the probatory sessions and await approval. Since this involved a waiting period of four to six weeks, patients with need of acute psychotherapy failed to receive the necessary treatment beyond being seen and assessed in probatory sessions.

Since the reform in 2017, all registered medical and psychological psychotherapists have been obliged to offer psychotherapeutic consultation times of 100 minutes within the period of a week. This ensured that patients with acute symptoms swiftly received an initial diagnosis and treatment recommendation (e.g., outpatient vs. inpatient treatment and/or psychopharmacotherapy). The other new reform element was the implementation of acute short-term psychotherapeutic interventions. According to this, immediately after offering patients 100 minutes of consultation, the psychotherapist was authorized to initiate an acute short-term psychotherapeutic intervention (maximum 24 sessions of 25 minutes each) (13), without having to apply for and await treatment approval by the SHI. All that needed to be done was to inform the SHI about the indication in the patient for such an intervention. If, in the therapist’s judgement, the patient needed short- or long-term psychotherapy additional to the acute intervention already given, as before he would have to seek the approval of the SHI.

The aim of the reform of the psychotherapy guidelines was to lower the currently high access threshold (14, 15) by making acute care an available route to outpatient psychotherapeutic treatment for all patients needing psychotherapy. Having to undergo pre-treatment evaluation over six sessions and then to await treatment approval for several weeks thereafter represent formidable barriers in general to anyone seeking therapy. While this applies to all patients with mental health issues, it might be especially true for patients with cMPs. Various studies demonstrate that patients with chronic somatic diseases have elevated prevalences of mental disorders compared to controls with no chronic somatic condition (16). This poses a serious healthcare problem, as they are often in particular need of treatment. Next to a significantly lower quality of life, increased morbidity and mortality rates, or overall higher treatment costs, the patient’s physical condition often deteriorates if the mental disorder remains untreated (17–19). Without a valid data basis to support this, patients with cMPs are often considered to be underrepresented in outpatient psychotherapy as are supposed to face specific barriers to receive (availability) and engage in (appropriate) psychotherapy (11). For example, results indicate that despite their increased need for care, patients with cMPs frequently experience worse access to psychotherapy as they are more likely to be unable to attend treatments due to their illness (15). Next to the overall intend of reduced waiting times and lower access barriers for outpatient psychotherapeutic treatment, the reform also intended to enable faster diagnostic clarification and possibly easier access to acute short-term psychotherapeutic interventions for all patients, and thereby, possibly making outpatient psychotherapy more accessible to patients with cMPs as well.

Due to the existing barriers for patients with cMPs to receive outpatient psychotherapy (11), we assume that the chances for this group have especially increased as a result of the psychotherapy reform.

Whether the reform has achieved its goal and whether the innovations introduced could therefore also serve as a model for care of patients with cMPs in other countries has been investigated in four sub-studies of the ES-RiP project (‘Evaluating effects of the structural reform of outpatient psychotherapy for patients with mental disorders in Germany – comparing patients with and without comorbid chronic physical condition’) (20). Results primarily focusing on substudies I, II and IV of the ES-RiP project can be found elsewhere (Kruse et al. unpublished^1^; 21–23). The current paper presents the major results of substudy III. As substudy III was very extensive we present here the initial results. Further findings will be reported separately. By analyzing routine data of the National Association of Statutory Health Insurance Physicians (KBV), the present study aimed to investigate (1.) the number of patients receiving outpatient psychotherapy between 2015 and 2019, (2.) whether the provision of mental health care had changed over time, (3.) whether there were discernible differences in psychotherapeutic care of patients MnoP versus patients with cMPs, and (4.) whether there were recognizable differences in the psychotherapeutic care of elderly patients (>50-79 years) as a group of patients with a high proportion of mental and physical comorbidity. As a special index group with an even higher rate of physical comorbidity and a general very low psychotherapeutic treatment rate (24), we also included (5.) the group of patients over 79 years of age in this substudy III of the ES-RiP project. The aim is to investigate whether the provision of mental health care had changed over time for this special group of patients.

## 2 Materials and methods

The ES-RiP project is based on a complete survey of outpatient psychotherapy services to patients including preparation and implementation of therapy for which medical and psychological psychotherapists submit their bills for reimbursement in Germany (20). We carried out a quantitative secondary analysis of data on psychotherapeutic care provision from 2015-2019 stored as routine data for the whole of Germany by the KBV. By this means, we attempted to clarify outpatient psychotherapeutic care as actually implemented at the level of treated patients. As the guideline reform took place in 2017, this year is considered a transitional year, precisely positioned in the middle of the investigation period. This enabled a pre-post analysis over the period from 2015 to 2019. Specifically, we compared the two years before and after the reform.

Due to data protection laws, the study team had no access to information on social data of patients and therapists, such as names and places of residence of the patients or locations of the therapists’ practices. Thus, we worked with completely anonymous data; nonetheless, approval was obtained from the Ethics Committee of the Justus Liebig University Giessen and Marburg – Faculty of Medicine (approval number: AZ 107/20; 6th October 2020). The Innovation Fund of the Federal Joint Committee of Germany grant number 01VSF19004 supported this work. The study is registered by Register-ID DRKS00020344.

### 2.1 Study sample

The KBV data comprises patient information such as billing data and diagnoses entered in the system by treating health care professionals (usually physicians and psychotherapists). Based on this information, we extricated our target sample of patients who are mentally ill.

Given the analyses are based on routine data, we attempted to achieve as much certainty about diagnoses documented in the system as possible by applying the M2F criterion. Patients were considered to be mentally ill if they had at least two documented mental diagnoses that were identical at a two-digit level (ICD-10: F30-F69) within 4 consecutive quarters. Therefore, the M2F criterion for a diagnosis was considered to be met, if the diagnosis had been coded as ‘confirmed’ in at least two different treatment cases within four consecutive quarters. Within the group of mentally ill patients, we further distinguished three sub-groups according to whether or not chronic somatic conditions were present in addition to mental disorders: (1.) patients with cMPs, (2.) MnoP, or (3.) a group of intermediate patients (neither meeting the criteria for cMPs nor MnoP). Somatic diagnoses were also confirmed applying the M2F criterion.

**Figure 1:**
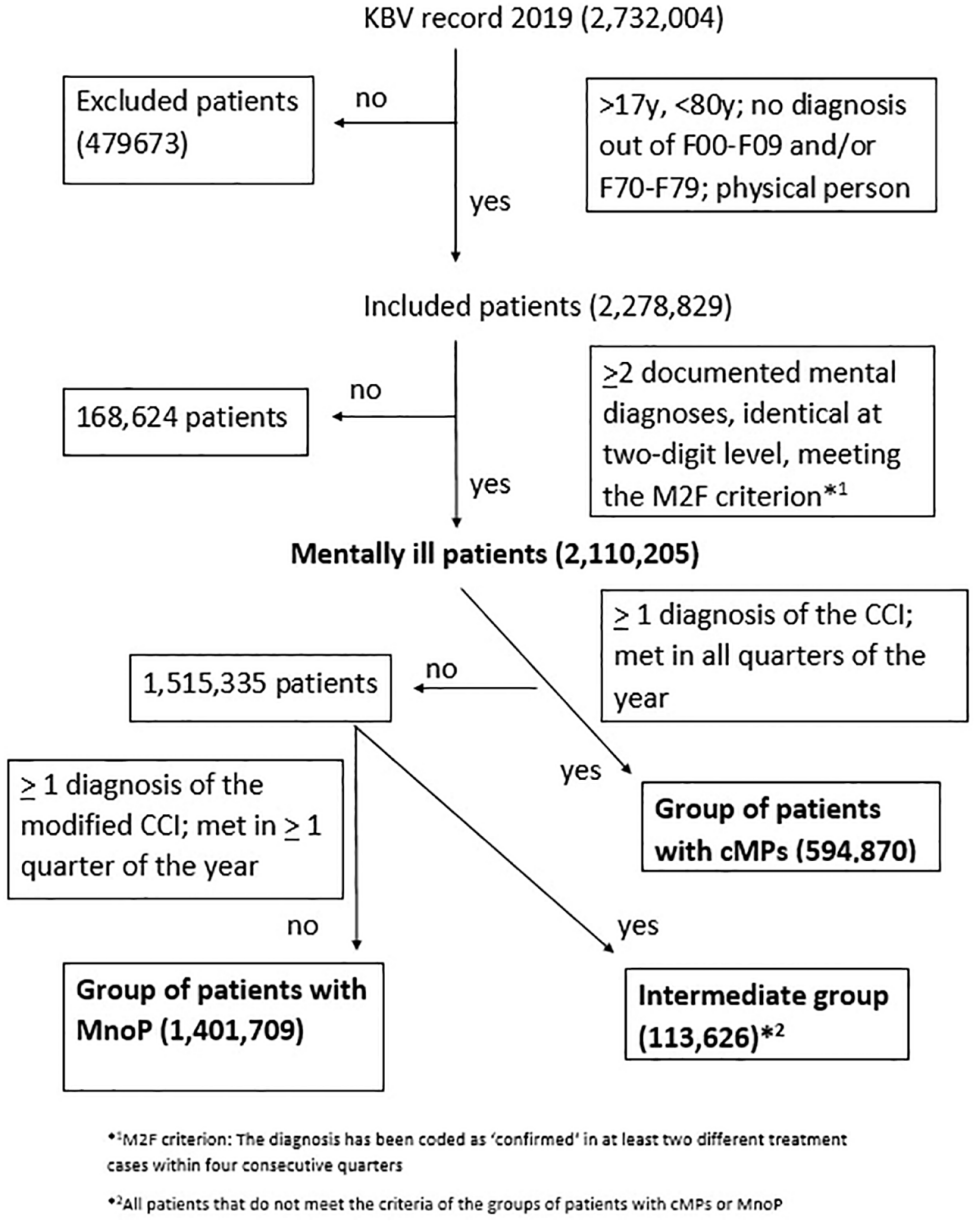
Flow chart illustrating the inclusion/exclusion of patients and the allocation to the three patient groups (cMPs, MnoP, intermediate group) for the year 2019.

A graphical overview of the inclusion and allocation of patients to the patient groups for 2019 is provided in **Figure 1**.

Exclusion criteria were: patients under the age of 18 and over 79 years of age; patients with a diagnosis from the ICD-10 chapter F00-09 (organic brain disorders) and/or F70-79 (intelligence impairment): for these patient groups, the indication for outpatient psychotherapy remains unclear and interactions between somatic and mental diseases can be assumed. For an overview see **Supplementary Table 2** in the **Supplementary Material**.

The group of patients over 79 years of age was primarily excluded in the ES-RiP project since the group remains largely untreated according to the literature (24). As there are some considerable results for the group of patients >79 years of age, data were analyzed in an auxiliary calculation. Therefore, only partial results for this group can be presented. The group of patients >79 years of age was not divided into sub-groups.

#### 2.1.1 Patients with cMPs

cMPs stands for the group of patients with a mental disorder who also have some chronic somatic disease. To ensure that chronic somatic diseases associated with high distress for the patient are included in the cMPs group, we used the Charlson Comorbidity Index (CCI) to define the presence of a chronic somatic disease (25, 26). The CCI is one of the most studied comorbidity indices designed to predict long-term mortality, and therefore, symptom burden. It includes 19 diseases, which are weighted based on their strength of their association with mortality (27). A recently published review provided the predictive power of the CCI for future morbidity and mortality risk (28), in a former review the test-retest reliability was found good, interrater reliability moderate to good (27). The diagnosis of a chronic somatic disease required an identical diagnosis according to the M2F criterion for at least one diagnosis of the CCI.

In addition to this general definition, according to a calendar year, we classified patients as patients with cMPs for a specific year when the above-mentioned criterion was met in all quarters of the calendar year.

#### 2.1.2 Patients with MnoP

We refer to patients with a mental disorder and no concurrent chronic physical condition as to patients with MnoP. The CCI lists only a selection of chronic physical conditions. To prevent the inclusion of patients with other chronic physical condition not listed in the CCI into the group of patients with MnoP, we used a modified version of the CCI (modCCI), based on the diagnoses of the CCI plus a list of chronic somatic diseases published as part of a Cochrane review (29) for this group. Accordingly, we considered patients with MnoP as patients who did not meet the M2F criterion for any disease listed in the modCCI in any quarter of the calendar year. **Supplementary Table 1** in the annex provides the list of all diagnoses of the modified CCI that were used in the ES-RiP study (20).

#### 2.1.3 Intermediate patients

The sub-group of intermediate patients included all patients in the group of mentally ill patients who could not be assigned to either the sub-group of patients with cMPs or to the sub-group of patients with MnoP in the four-quarter period under review. These included, for example, patients who had two confirmed identical diagnoses of a somatic disease from the modified CCI, which is not represented in the CCI. These were therefore patients who were too physically stressed to be assigned to MnoP but were not physically stressed enough to be assigned to cMPs.

### 2.2 Statistical analyses

Since the data originate from a complete survey of mental health care provision in Germany, analyses are limited to descriptive statistics. For separate patient groups and specific services, we calculated the absolute frequencies in the respective years 2015 to 2019, absolute and relative changes of these frequencies as well as the percentage of separate subgroups of patients or services respectively, and the absolute changes in these percentages in each of the years 2015 to 2019. Patients with cMPs, with MnoP and intermediate patients represent the three ‘diagnosis groups’ in this study. Additionally, we analyzed data for the group of patients >79 years of age in an auxiliary calculation, which included the calculation of absolute frequencies for the years 2015 to 2019 as well as the absolute and relative changes in these frequencies.

To answer the research questions, three target values were considered: the number of patients who received at least one outpatient psychotherapeutic service during the study period and the number of outpatient psychotherapeutic services billed per diagnosis group per year. In addition, the age and sex distribution of the treated patients was examined.

## 3 Results

**Figure 2:**
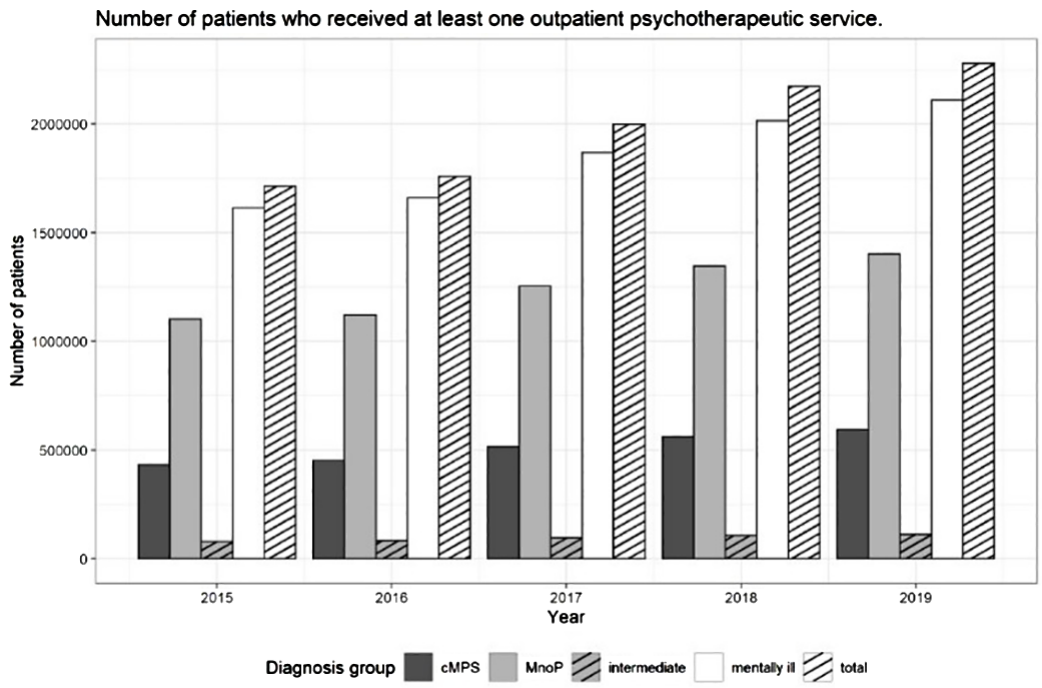
Number of patients who received at least one outpatient psychotherapeutic service in a specific diagnosis group between 2015 and 2019 – differentiated by diagnosis group.

The size of the patient collective as well as the number of patients in the individual sub-groups increased steadily over time (**Figure 2**, data in **Supplementary Table 3** in the **Supplementaary Material**). The group ‘KBV record’ comprises all patients who had received at least one outpatient psychotherapeutic service in the year referred to (this corresponds to the line ‘KBV data set’ in **Supplementary Table 2**, for example, 2,732,004 in 2019).

**Figure 3:**
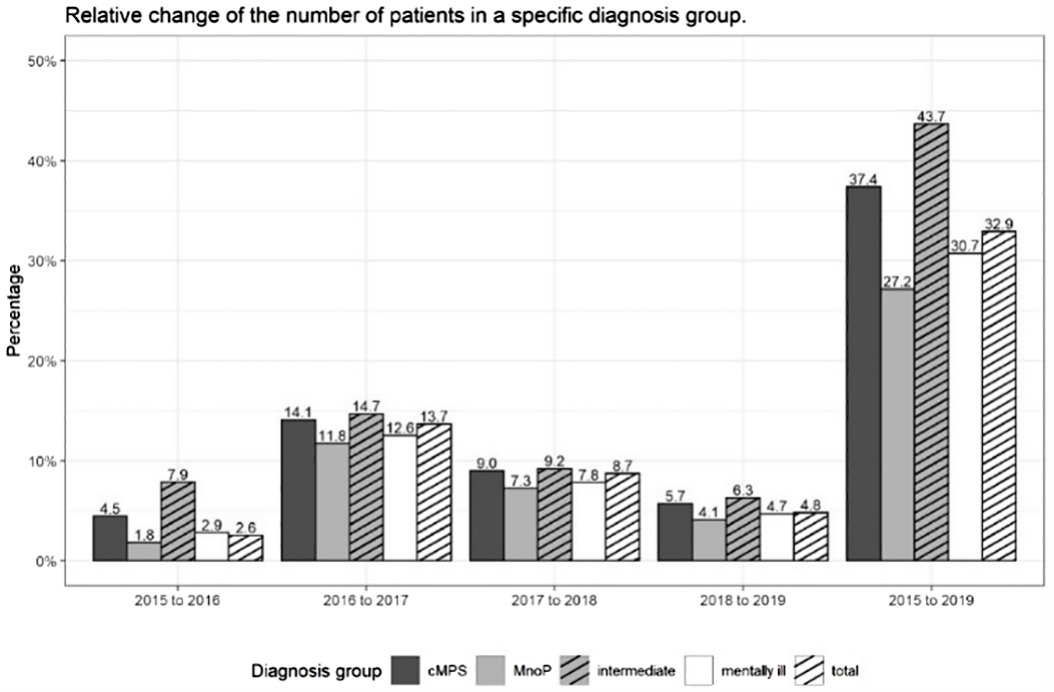
Relative changes in the number of patients receiving at least one outpatient psychotherapeutic service in a specific diagnosis group compared to the previous year or 2015 in percent - differentiated by diagnosis group.

In the KBV record, the number of data entries rose from 2,006,249 in 2015 to 2,732,004 in 2019 (change 36.2%), the number of included patients increased from 1,714,777 in 2015 to 2,278,829 in 2019 (change: 32.9%). The total number of mentally ill patients added up to 1,614,458 in 2015 and to 2,110,205 in 2019 (change: 30.7%). For 2019, out of the group of mentally ill patients we could assign 594,870 patients to the group of patients with cMPs (change compared to 2015: 37.4%), 113,626 to the group of intermediate patients (change compared to 2015: 43.7%) and 1,401,709 to the group of patients with MnoP (change compared to 2015: 27.2%). For percentage changes regarding included patients and patient subgroups, please refer to **Figure 3**.

From 2015 to 2019, the number of patients with cMPs showed a larger increase (37.4%) than the number of patients with MnoP (27.2%). The number of patients who could not be clearly assigned to a sub-group increased the most (43.7%); however, as described above, the absolute frequency of these patients was very low. The largest increases took place from 2016 to 2017 (percentage changes: cMPs 14.1%; MnoP 11.8%; intermediate 14.7%) and then from 2017 to 2018 (percentage changes: cMPs 9.0%; MnoP 7.2%; intermediate 9.3%). For details, see also **Figure 3** and **Supplementary Table 3** in the **Supplementary Material**.

**Figure 4:**
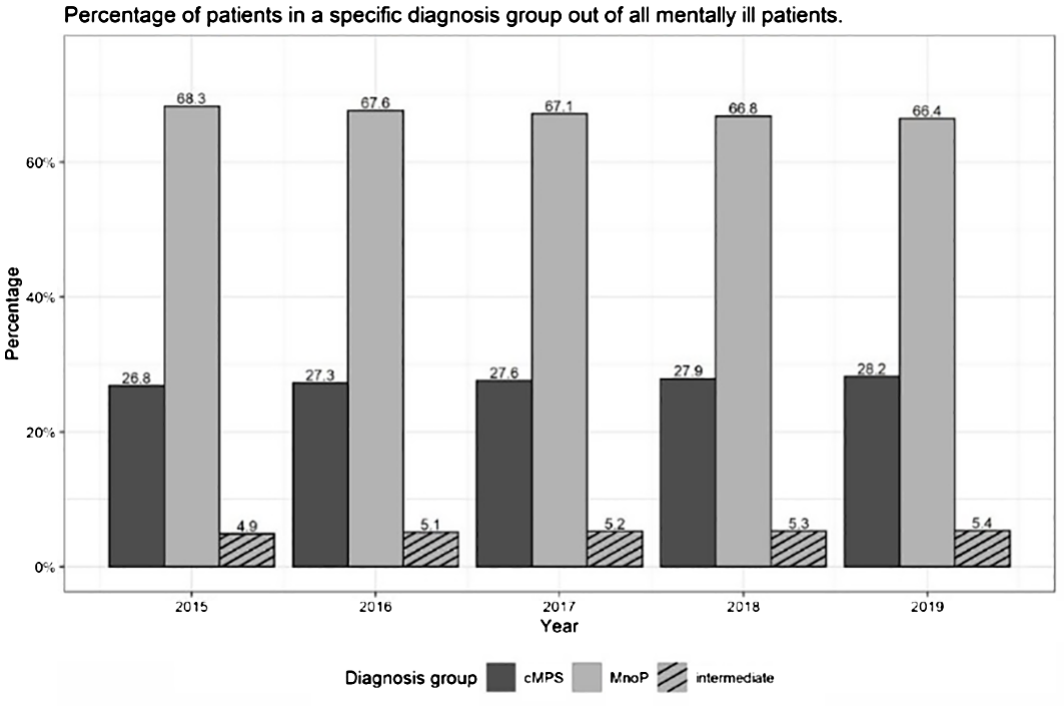
Percentage of patients who received at least one outpatient psychotherapeutic service in a specific diagnosis group in the group ‘mentally ill’ - differentiated by diagnosis group.

In 2015, 26.8% of the mentally ill patients who got treatment met the criteria of patients with cMPs and 68.3% the criteria of patients with MnoP. The percentages of these two diagnosis groups remained relatively stable but were constantly and slightly shifting in favor of the cMPs group over time. The percentage of patients with cMPs increased from 26.8% to 28.2% (+1.4%) between 2015 and 2019, the percentage of patients with MnoP decreased from 68.3% to 66.4% (-1.9%) between 2015 and 2019. The increases and decreases occurred evenly over the observation period. The remaining 5% of the mentally ill who could not be assigned either to cMPs or MnoP represent the patients of the intermediate group. This proportion hardly changed over the study period. For details, see **Figure 4** and **Table 4** in the **Supplementary Material**.

The results at the level of the billed psychotherapy services essentially reflected the previously reported results of the patient collective. The number of outpatient psychotherapeutic services increased significantly for patients in both cMPs and MnoP groups from 2015 to 2019 (see **Supplementary Material Table 5**, **Supplementary Figure 1**). The largest increases took place from 2016 to 2017 and from 2017 to 2018.

The number of services billed for patients with cMPs increased slightly more (43.4%) than the number of services billed for patients with MnoP (35.1%) (see **Supplementary Material Figure 2, Supplementary Table 5**).

Within the group of mentally ill patients, around 25% of the services were provided for patients with cMPs and around 70% for patients with MnoP. Only a small proportion of 5% of the services were billed for intermediate patients with mental diseases. Over time, the proportion of services billed for cMPs increased slightly by about 1% and the proportion of services billed for MnoP decreased by about 1.5%. These minimal shifts in percentages occurred evenly over time. For details see **Supplementary Material Table 6** and **Supplementary Figure 3**.

**Figure 5:**
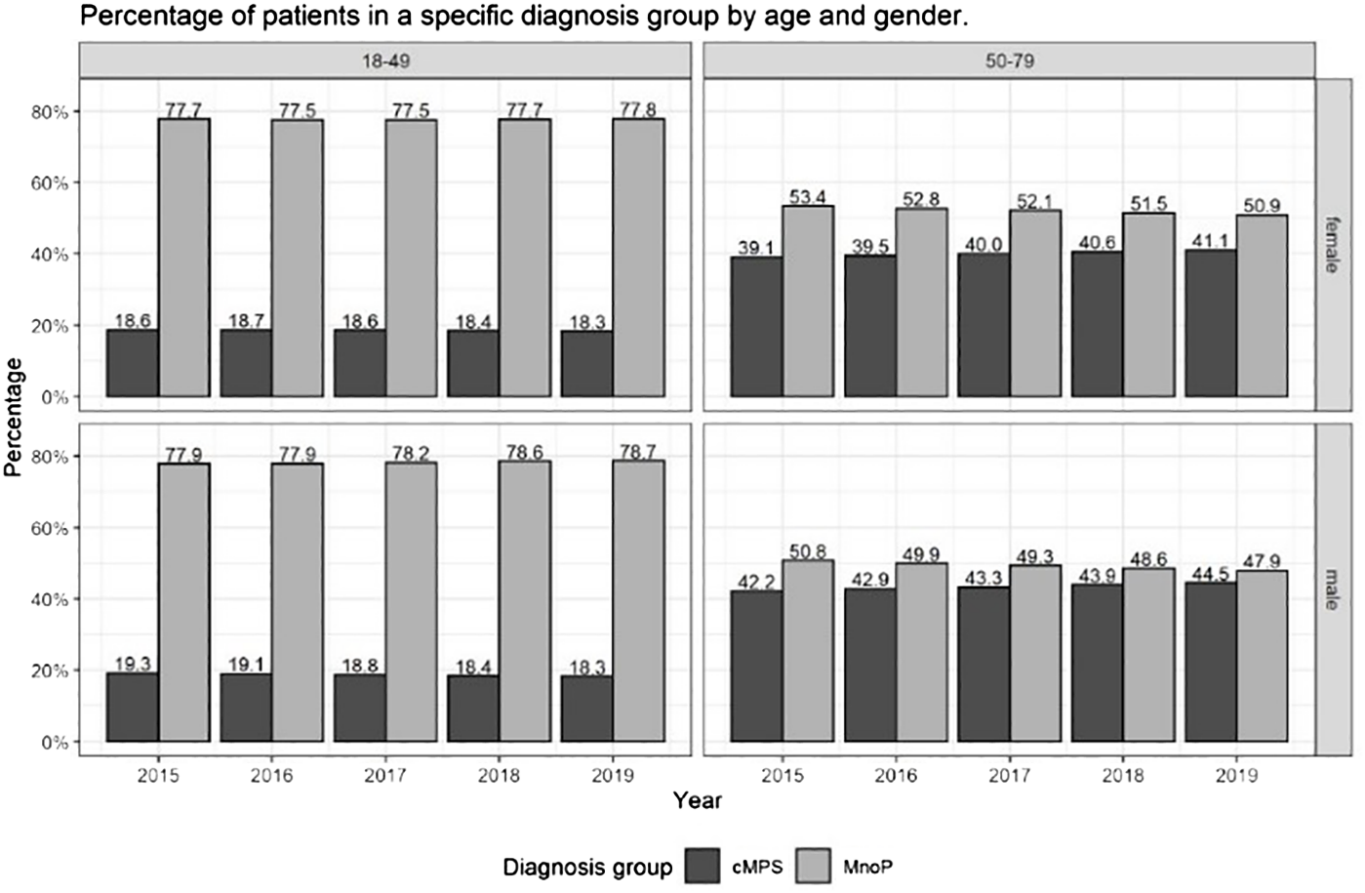
Percentage of patients who received at least one outpatient psychotherapeutic service in a specific diagnosis group in the group ‘mentally ill’ patients - differentiated by age and sex.

**Figure 5** and **Supplementary Table 7** in the **Supplementary Material** show the percentage of patients in each diagnosis group in relation to all treated patients who had received at least one psychotherapeutic service. The figure sums up the individual age cohorts into two groups of 18-49 years and 50-79 years, designing two age groups that cover a similar age range. In addition, the group of patients > 50 years of age is of interest because studies show that due to the comorbidity of mental disorders and chronic physical pain, patients > 50 years of age incur higher healthcare costs and have a lower quality of life (9, 10). The absolute frequencies of the included patients as well as the percentage change in each year relative to the previous year and percentage change in 2019 relative to 2015 – differentiated by age in 10-year cohorts, sex, and diagnosis group – can be found in **Supplementary Table 8** in the **Supplementary Material**.

**Figure 5** shows that the percentages of patients with cMPs and MnoP vary considerably with age. Among the 18-49-year-old patients, the percentage of people with cMPs remained relatively constant at just under 19% over the years of the observation period. The percentage was slightly higher for men than for women in 2015 (19.3% versus 18.6%). The percentage of patients with MnoP showed a similar trend. For women under the age of 49, it remained almost unchanged from 2015 to 2019 at around 77.7%. For men under the age of 49, it was 77.9% in 2015 and 2016 and rose minimally to 78.6% in 2018 and 78.7% in 2019.

Among the patients over 50 years of age, the differences in the percentages between men and women as well as the change of the percentages over time were bigger: the percentage of men with cMPs among all men with mental disorders increased continuously from 42.2% in 2015 to 44.5% in 2019 whereas the percentage of men with MnoP fell continuously from 50.8% in 2015 to 47.9% in 2019. The percentage of women with a cMPs increased continuously from 39.1% in 2015 to 41.1% in 2019 while the percentage of women with MnoP dropped continuously from 53.4% in 2015 to 50.9% in 2019. In summary, for people over the age of 50 with mental disorders, the percentage of patients with cMPs was at least three percent higher in men than in women in each calendar year between 2015 and 2019. The percentage of patients with cMPs increased by around 2% between 2015 and 2019 in both men and women (for details **Supplementary Material Table 8**). We also see a particularly high growths in the total number of mentally ill patients in the group of patients aged 50-79 years added up to 572,207 in 2015 and 820,907 in 2019 (change: +43,5%) in comparison to the group of patients aged 18-49 years added up to 1,003,134 in 2015 and 1,232,036 in 2019 (change: + 22,8%) (for details **Supplementary Material Table 8**).

**Supplementary Figure 4** and **Suppplementary Table 9** in the **Supplementary Material** summarize the percentages of services that are attributable to the respective diagnosis groups, summed up into two age groups of 18-49 years and 50-79 years. All differences in the diagnostic groups differentiated by age and sex and all changes between 2015 and 2019 were identical to those at the level of the patients. However, the percentage of services for patients with cMPs was almost 1% lower than the percentages of the corresponding patient groups among the under 49-year-old people and around 2% lower than the percentages of the corresponding patient groups among the >50-year-old patients.

The findings on the group of >79-year-old patients were the most surprising of all: while 52,023 people >79 years of age received at least one outpatient psychotherapeutic service in Germany in 2015, it was 134,455 in 2019 (**Supplementary Table 10**). This represents an increase of 158.4%, far exceeding the absolute increases in all age groups.

## 4 Discussion

By means of a quantitative secondary analysis of data on psychotherapeutic care provision from 2015-2019 stored as routine data for the whole of Germany by the KBV, we attempted to clarify outpatient psychotherapeutic care as actually implemented at the level of treated patients. In 2017 a structural reform of the German outpatient psychotherapy guideline was carried out. It aimed to lower the currently high access threshold to outpatient treatment by reducing waiting times and improving access to psychotherapy. In summary, the total number of mentally ill patients added up to 1,614,458 in 2015 and 2,110,205 in 2019 (change: +30.7%). This increase corresponds to the existing social trend towards increasing acceptance and increasing use of psychotherapy in Germany (30). For 2019 – in comparison to 2015 – out of the group of mentally ill patients we could assign 594,870 patients to the group of patients with cMPs (change +37.4%), 113,626 to the group of intermediate patients (change +43.7%), 1,401,709 to the group of patients with MnoP (change +27.2%). The percentage of patients with cMPs of all mentally ill people who received services increased by +1.4% whereas the percentage of patients with MnoP decreased by 1.9%. The results at the level of the billed psychotherapy services essentially reflected the results of the patient collective.

The number of psychotherapists had only increased by 19% (paper in preparation) over these five years and the increase in the absolute frequencies was particularly strong in the period of the reform (2016 to 2017 as well as 2017 to 2018). Therefore, the increase of the total number of mentally ill patients who received at least one outpatient psychotherapeutic service in a calendar year can be considered as an effect of the newly introduced ‘consultation hours’ and the option of an ‘acute short-term psychotherapeutic intervention’ as elements of the structural reform of the SHI.

A direct comparison with the epidemiological burden of a comorbidity of chronic physical conditions and mental disorders in the general population is not possible. As far as we know there is no study giving exact prevalences for Germany. But a high epidemiological burden can be estimated: One third of all hospital patients with primarily somatic problems show pathological psychological symptoms (31). At the same time, the prevalence of chronic somatic diseases is increased in the presence of mental disorders, especially in the context of depression (32). Even patients with somatoform disorders find it difficult to find outpatient psychotherapeutic treatment (33), so it is hypothesized that patients with an actual physical comorbidity have even more difficult access. Mack et al. (34) also describe a care deficit for patients who “only” have a comorbidity of exclusively mental disorders. We therefore assumed that patients with cMPs in particular would benefit from easier access to psychotherapy.

Access to psychotherapeutic services seems to be slowly improving for patients with cMPs. This is suggested by discernible differences in the care for patients with MnoP versus patients with cMPs that have occurred over the time period of five years investigated, as determined by the reimbursement bills submitted to the KBV for psychotherapeutic services under the new billing codes. However, since the increases and decreases in the percentages took place evenly over the individual years, it is not possible to conclude that the reform of the psychotherapy guidelines had a direct and decisive influence on these changes.

The increase in care for patients with cMPs is particularly high in the group of patients aged 50-79 years. We also see a particularly high growths in the total number of mentally ill patients in the group of patients aged 50-79 years (change: +43,5%) in comparison to the group of patients aged 18-49 years (change: + 22,8%) from 2015 to 2019. The increase in care of patients with cMPs could accordingly be related to an overall change in the age distribution of patients who have access to psychotherapy with an older age being associated with a higher rate of physical comorbidities. At the same time, however, the proportion of patients with cMPs in the 50–79-year-old group is increasing, too, which illustrates the improved access to psychotherapy for this group of patients.

An unexpected result is the disproportionate increase in psychotherapeutically treated patients >79 years of age between 2015 and 2019. The 158.4% increase could be associated with a trend, which Schneider et al. already predicted in 2000, 2003 and 2004 (35–37): The cohorts now entering the group of the elderly are actively seeking psychotherapy to a far greater extent than adults after the Second World War. This can be mostly attributed to the elderly having already experienced the helpful effect of psychotherapy themselves or in others in their environment. In this respect, it is more important than ever to question the prejudice on the part of psychotherapists that older people are very skeptical about psychotherapy. However, despite this increase in the proportion of patients >79 years of age in 2019, only 0.9% of the patients included in this study were in this age group compared to all included patients, while 5.7 million people >79 years lived in Germany in 2019 (7% of the total population) (2015: 4.7 million, corresponding to 6% of the total population) (38). In this respect, there is still an ‘indication censorship’ for psychotherapy regarding elderly people (24). Intuitively, one would rather assume that those patients with cMPs, especially older patients, have a greater need for psychotherapeutic services. However, the fact that they receive fewer benefits requires further investigation. The question is whether these are phenomena of self-transference (39) and ageism (40) by (often younger) psychotherapists towards their older patients. Negative stereotypes of older people among the therapists not only lead to a less frequent recognition of an indication for psychotherapy, but also to fewer offers of services. In this context, it is worth raising the question whether more limited but realistic therapy goals could be defined for older people with shorter treatments. The negative attitudes towards the psychotherapeutic treatment options for older people, which may be unconscious to the therapists themselves, can only be changed during the training period through professional training under supervision.

In any case, we agree with Charlson and Wells (26) that, regardless of age, there is a need to meet the ‘challenge of comorbidity in this decade’. This is especially true of the cohort of older people with mental disorders and comorbid chronic physical conditions. “The goal of personalized medicine requires an approach that ‘integrates social, psychological, ecological and basic biological information to tailor the best approach’” (27, p.149). The prerequisite for achieving this goal is the expertise of highly trained and experienced clinicians.

### 4.1 Limitations

Since the data for this analysis are based on data on psychotherapeutic care provision from 2015-2019 stored as routine data for the whole of Germany by the KBV, we could only include patients who received at least one outpatient psychotherapeutic service in a calendar year. The percentage of patients in the cMPs group who had not received any outpatient psychotherapeutic services cannot be calculated from the KBV data set. Additionally, the data set only contains patients for whom services were actually billed in the statutory health insurance system. The perspective of psychotherapists, possible differences in and effects of the frequency of psychotherapeutic offers, variability across psychotherapists and therapeutic settings as well as regional impacts were also not part of this analysis.

The KBV data underlying the analysis were collected as administrative, not clinical, data. We attempted to achieve as much certainty about diagnoses documented in the system as possible by applying the M2F criterion. Nevertheless, the administrative origin of data may cause validity issues, which can arise, for example, from transferring diagnoses from one quarter to the next without checking the diagnosis again. Such errors cannot be traced in the retrospectively evaluated KBV data.

Due to data protection regulations, the data used could not be evaluated in combination with sociodemographic variables. This was dictated by the SHI.

## 5 Conclusion

Looking at the growing number of patients receiving outpatient psychotherapy and of billed psychotherapeutic services, access to psychotherapeutic services appears to have been facilitated for all patients by the 2017 guideline reform. However, the proportion of patients with cMPs increased evenly year by year, while the proportion of patients with MnoP showed a continuous decline. It is therefore not possible to conclude that the reform of the psychotherapy guidelines has had a direct influence on these changes. The strength of the ES-RiP project lies in a triangulation of different data sources to investigate pre-reform to post-reform changes. For a further conclusion in terms of the research question, an analysis of data from the patients’ as well as from the providers’ perspective will be carried out in the future.

## Data availability statement

The original contributions presented in the study are included in the article/**Supplementary Material**. Further inquiries can be directed to the corresponding author.

## Ethics statement

The studies involving human participants were reviewed and approved by the Ethics Committee of the Justus Liebig University Giessen and Marburg – Faculty of Medicine (approval number: AZ 107/20; 6th October 2020). Written informed consent was not required for the secondary analysis of anonymized data. The studies were conducted in accordance with the local legislation and institutional requirements.

## Author contributions

JJ: Writing – original draft, Writing – review & editing. HK: Writing – original draft, Writing – review & editing. AC: Methodology, Writing – review & editing. CS: Data curation, Formal analysis, Methodology, Writing – review & editing. GH: Conceptualization, Funding acquisition, Project administration, Supervision, Writing – review & editing. HF: Conceptualization, Funding acquisition, Project administration, Supervision, Writing – review & editing. JK: Conceptualization, Funding acquisition, Project administration, Supervision, Writing – review & editing.

## Footnotes

1 Kruse J, Kampling H, Filali Bouami S, Grobe TG, Hartmann M, Jedamzik J, et al. Ambulante psychotherapeutische Versorgung in Deutschland – Evaluation der Strukturreform. *Dtsch Arztebl Int*. (Unpublished).
