# Supplementary material for: Do the elderly and those with comorbid chronic physical conditions have improved access to outpatient psychotherapy post structural reforms in Germany? Results of the ES-RiP study
Source: Front Psychiatry. 2024 Apr 29;15:1349603. doi: 10.3389/fpsyt.2024.1349603 (PMC11090099; doi:10.3389/fpsyt.2024.1349603)
Supplement: Supplementary file 1 [file DataSheet_1.docx]

**Suppl. Figures 1-4 (supplemental material)**

**Suppl. Fig. 1. Number of outpatient psychotherapeutic services that were billed for specific diagnosis groups between 2015 and 2019.**

**
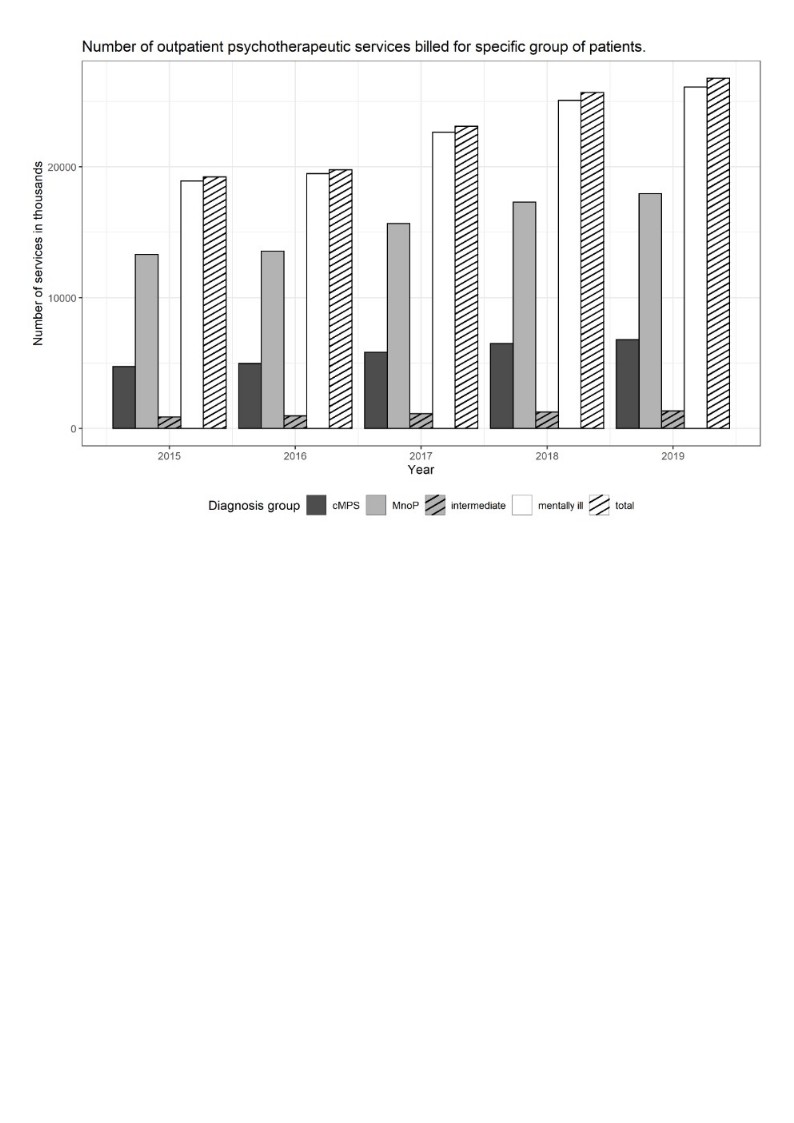
**

**Suppl. Fig. 2. Relative changes in the number of outpatient psychotherapeutic services that were billed for a specific diagnosis group between 2015 and 2019 compared to the previous year or 2015 in percent - differentiated by diagnosis group.**

**
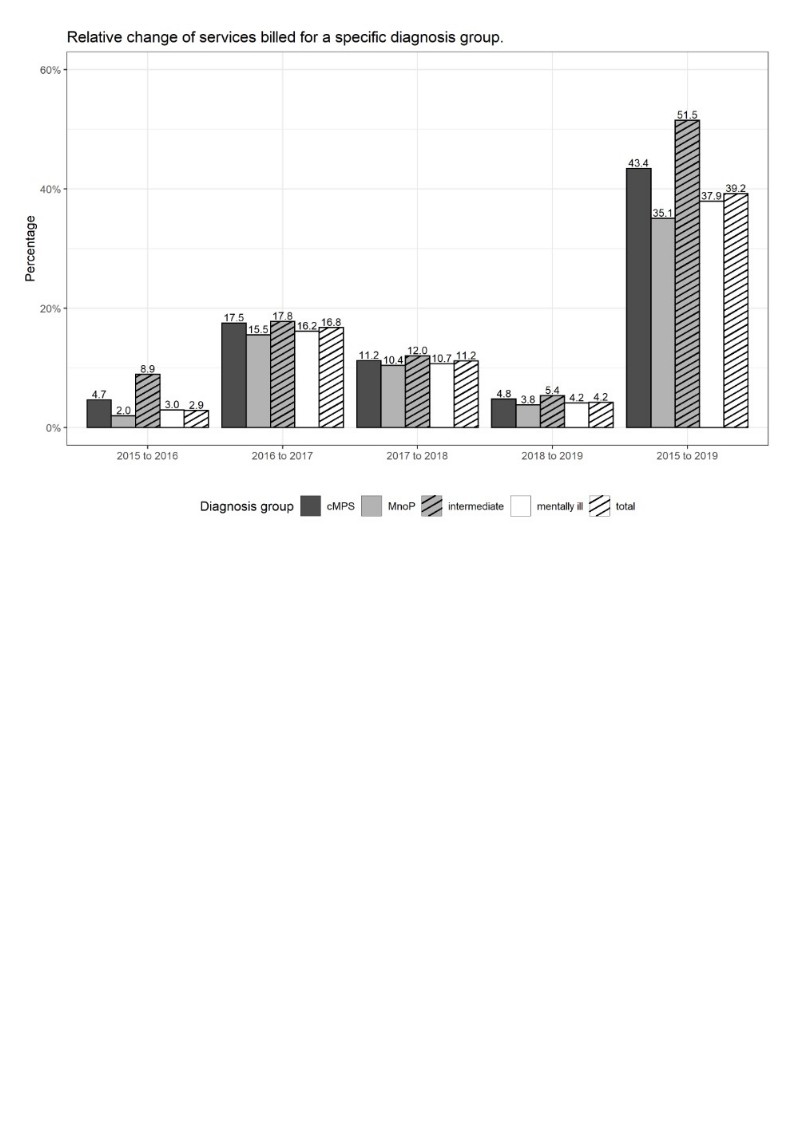
**

**Suppl. Fig. 3. Percentage of services that were billed for the individual diagnosis groups of all services that were billed for mentally ill people - differentiated by diagnosis group.**

**
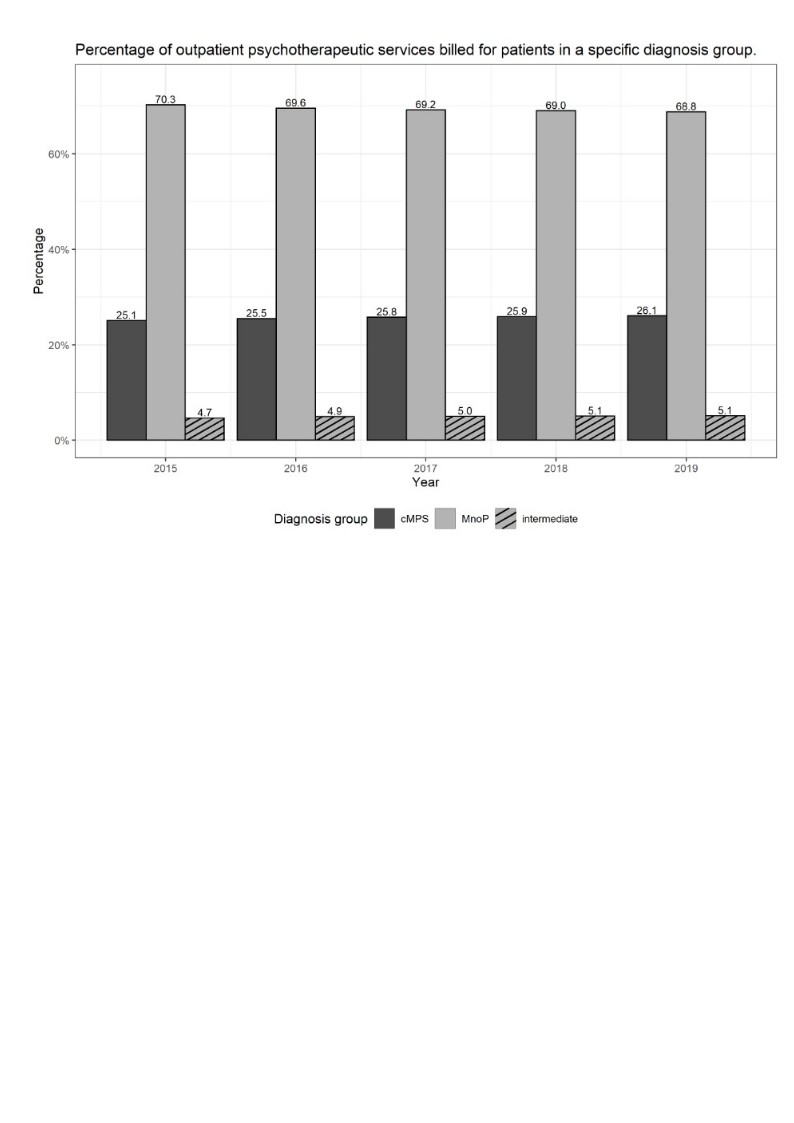
**

**Suppl. Fig. 4. Percentage of services billed for the individual diagnosis groups of all services that were billed for mentally ill people - differentiated by age and sex of the patients.**

**
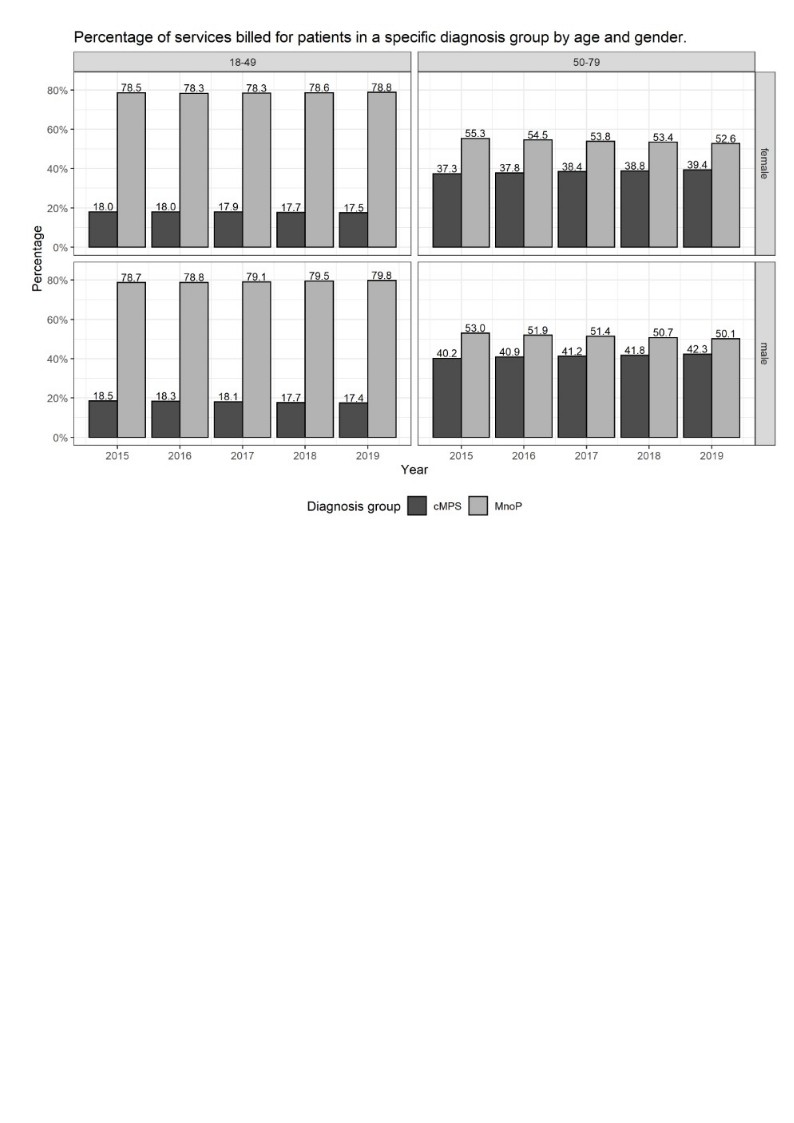
**

**Suppl. Tables 1-10 (supplemental material)**

**Suppl. Tab. 1. Modified Charlson Comorbidity Index (modCCI) including ICD-10 codes**

| **ICD-10 Code** | **Chronic disease** | **ICD-10 Code** | **Chronic disease** |
| --- | --- | --- | --- |
| B18 | Chronic viral hepatitis | I86.4 | Gastric varices |
| B20-B24 | Human immunodeficiency virus [HIV] disease | I98.2 | Oesophageal varices without bleeding in diseases classified elsewhere |
| C00-C97 | Malignant neoplasms | J40-J47 | Chronic lower respiratory diseases |
| D86 | Sarcoidosis | J60-J70 | Lung diseases due to external agents |
| E10-E14 | Diabetes mellitus | K25 | Gastric ulcer |
| E66.02/.12/  .22/.82/.92 | Any obesity: WHO grade III | K26 | Duodenal ulcer |
| G04.1 | Human T-cell lymphotropic virus-associated myelopathy | K27 | Peptic ulcer. site unspecified |
| G10-G12 | Systemic atrophies primarily affecting the central nervous system (Huntington disease. Hereditary ataxia. Spinal muscular atrophy and related syndromes) | K28 | Gastrojejunal ulcer |
| G20-G22 | Parkinson disease and Parkinsonism | K50 | Crohn’s disease |
| G23 | Other degenerative diseases of basal ganglia | K51 | Ulcerative colitis |
| G25 | Other extrapyramidal and movement disorders | K55.1 | Chronic vascular disorders of intestine |
| G35 | Multiple sclerosis | K55.8. K55.9 | Other vascular disorders of intestine and unspecified vascular disorder of intestine |
| G40 | Epilepsy | K70-K77 | Liver diseases |
| G43 | Migraine | K86.1 | Other chronic pancreatitis |
| G45 | Transient cerebral ischaemic attacks and related syndromes | L93 | Lupus erythematosus |
| G46 | Vascular syndromes of brain in cerebrovascular diseases | M05-M14 | Inflammatory polyarthropathies |
| G62. G63 | Polyneuropathy | M30-M36 | Systemic disorders of connective tissue |
| G70-G73 | Diseases of myoneural junction and muscle | M79.7 | Fibromyalgia |
| G80.1 | Spastic diplegic cerebral palsy | N03 | Chronic nephritic syndrome |
| G80.2 | Spastic hemiplegic cerebral palsy | N04 | Nephrotic syndrome |
| G81 | Hemiplegia | N05.2 | Diffuse membranous glomerulonephritis |
| G82 | Paraplegia and tetraplegia | N05.3 | Diffuse mesangial proliferative glomerulonephritis |
| G83 | Other paralytic syndromes | N05.4 | Diffuse endocapillary proliferative glomerulonephritis |
| G90.5/.6 | CRPS type I & II | N05.5 | Diffusec glomerulonephritis |
| H34.0 | Transient retinal artery occlusion | N05.6 | Dense deposit disease |
| H35.3 | Macular degeneration | N05.7 | Diffuse crescentic glomerulonephritis |
| I09.9 | Unspecified rheumatic heart disease | N18 | Chronic kidney disease |
| I11.0, I12.0, I13.0-.2 | Hypertensive heart and or renal diseases with heart and or renal failure | N19 | Unspecified kidney failure |
| I20-I25 | Ischaemic heart diseases | N25.0 | Renal osteodystrophy |
| I27 | Other pulmonary heart diseases | N30.2 | Chronic cystitis |
| I42, I43 | Cardiomyopathy and cardiomyopathy in diseases classified elsewhere | N41.1 | Chronic prostatitis |
| I50 | Heart failure | N80 | Endometriosis |
| I60-I69 | Cerebrovascular diseases | P29.0 | Neonatal cardiac failure |
| I70 | Atherosclerosis | U55 | Registration for organ transplant |
| I71 | Aortic aneurysm and dissection | Z49.0 | Preparatory care for dialysis |
| I73.1 | Thromboangiitis obliterans [Buerger] | Z49.1 | Extracorporeal dialysis |
| I73.8/.9 | Other specified peripheral vascular diseases and unspecified peripheral vascular disease | Z49.2 | Other dialysis |
| I77.1 | Stricture of artery | Z94.0 | Kidney transplant status |
| I79.0 | Aneurysm of aorta in diseases classified elsewhere | Z94.4 | Liver transplant status |
| I79.2 | Peripheral angiopathy in diseases classified elsewhere | Z95.8 Z95.9 | Presence of other cardiac and vascular implants and grafts and presence of cardiac and vascular implant and graft. Unspecified |
| I85.0 | Oesophageal varices with bleeding | Z99.2 | Dependence on renal dialysis |
| I85.9 | Oesophageal varices without bleeding |  |  |

**Suppl. Tab. 2. Absolute frequencies of patients who received at least one outpatient psychotherapeutic service between 2015 and 2019, as well as percentage change in each year relative to the previous year and percentage change in 2019 relative to 2015 - differentiated according to inclusion and exclusion criteria.**

| Recipient of service | Absolute frequencies  2015 | Change  2015-2016  in % | Absolute frequencies  2016 | Change  2016-2015 in % | Absolute frequencies 2017 | Change  2017-2018 in % | Absolute frequencies 2018 | Change  2018-2019 in % | Absolute frequencies 2019 | Change  2015-2019 in % |
| --- | --- | --- | --- | --- | --- | --- | --- | --- | --- | --- |
| <18 years | 237,927 | 2.40 | 243,629 | 22.18 | 297,661 | 16.43 | 346,555 | 7.27 | 371,745 | 56.24 |
| >79 years | 8,524 | 13.14 | 9,644 | 33.69 | 12,893 | 31.28 | 16,926 | 18.60 | 20,074 | 135.50 |
| F00-09 | 39,631 | 2.85 | 40,761 | 16.46 | 47,470 | 9.20 | 51,836 | 5.41 | 54,641 | 37.87 |
| F70-79 | 13,058 | 1.72 | 13,283 | 20.82 | 16,048 | 15.40 | 18,520 | 7.80 | 19,964 | 52.89 |
| Excluded*² | 291,472 | 2.85 | 299,789 | 21.48 | 364,194 | 15.78 | 421,659 | 7.47 | 453,175 | 55.48 |
| Included | 1,714,777 | 2.56 | 1,758,692 | 13.67 | 1,999,107 | 8.73 | 2,173,660 | 4.84 | 2,278,829 | 32.89 |
| Mentally ill | 1,614,458 | 2.85 | 1,660,488 | 12.55 | 1,868,895 | 7.85 | 2,015,518 | 4.70 | 2,110,205 | 30.71 |
| KBV record*¹ | 2,006,249 | 2.60 | 2,058,481 | 14.81 | 2,363,301 | 9.82 | 2,595,319 | 5.27 | 2,732,004 | 36.17 |

*¹ Patients listed as separate persons in the KBV dataset but do not have to correspond to a physical person.

*² Subsumes patients who meet the exclusion criteria (<18 years, >79 years, F00-09, F70-79) and data entries that do not correspond to a physical person.

**Suppl. Tab. 3. Absolute frequencies (a. f.) of patients who received at least one outpatient psychotherapeutic service in a specific diagnosis group between 2015 and 2019, as well as percentage change (p. c.) in each year relative to the previous year and percentage change in 2019 relative to 2015 - differentiated by diagnosis groups.**

| Diagnosis group | a. f. 2015 | p. c. 2015-2016 | a. f. 2016 | p. c. 2016-2017 | a. f. 2017 | p. c. 2017-2018 | a. f. 2018 | p. c. 2018-2019 | a. f. 2019 | p.c. 2015-2019 |
| --- | --- | --- | --- | --- | --- | --- | --- | --- | --- | --- |
| cMPs | 433,067 | 4.49 | 452,507 | 14.09 | 516,244 | 9.00 | 562,689 | 5.72 | 594,870 | 37.36 |
| MnoP | 1,102,302 | 1.85 | 1,122,651 | 11.77 | 1,254,783 | 7.26 | 1,345,938 | 4.14 | 1,401,709 | 27.16 |
| Intermediate patients | 79,089 | 7.89 | 85,330 | 14.69 | 97,868 | 9.22 | 106,891 | 6.30 | 113,626 | 43.67 |
| Mentally ill | 1,614,458 | 2.85 | 1,660,488 | 12.55 | 1,868,895 | 7.85 | 2,015,518 | 4.70 | 2,110,205 | 30.71 |
| Included patients | 1,714,777 | 2.56 | 1,758,692 | 13.67 | 1,999,107 | 8.73 | 2,173,660 | 4.84 | 2,278,829 | 32.89 |

**Suppl. Tab. 4. Percentage of mentally ill patients who received services in a specific diagnosis group and differences in these proportions in each year between 2015 and 2019 as well as percentage change (p. c.) in each year relative to the previous year - differentiated by diagnosis groups.**

| Diagnosis group | Percentage 2015 | Difference 2015-2016 in % | Percentage 2016 | Difference 2016-2017 in % | Percentage 2017 | Difference 2017-2018 in % | Percentage 2018 | Difference 2018-2019 in % | Percentage 2019 | Difference 2015-2019 in % |
| --- | --- | --- | --- | --- | --- | --- | --- | --- | --- | --- |
| cMPs | 26.82 | 0.43 | 27.25 | 0.37 | 27.62 | 0.29 | 27.92 | 0.27 | 28.19 | 1.37 |
| MnoP | 68.28 | -0.67 | 67.61 | -0.47 | 67.14 | -0.36 | 66.78 | -0.35 | 66.43 | -1.85 |
| Intermediate patients | 4.90 | 0.24 | 5.14 | 0.10 | 5.24 | 0.07 | 5.30 | 0.08 | 5.38 | 0.49 |

**Suppl. Tab. 5. Absolute frequencies (a. f.) of outpatient psychotherapeutic services that were billed for a specific diagnosis group in each year from 2015 to 2019, as well as percentage change (p. c.) in each year relative to the previous year and percentage change (p.c.) in 2019 relative to 2015 – differentiated by diagnosis groups.**

| Diagnosis group | a. f. 2015 | p. c. 2015-2016 | a. f. 2016 | p. c. 2016-2017 | a. f. 2017 | p. c. 2017-2018 | a. f. 2018 | p. c. 2018-2019 | a. f. 2019 | p. c. 2015-2019 |
| --- | --- | --- | --- | --- | --- | --- | --- | --- | --- | --- |
| cMPs | 4,744,238.5 | 4.68 | 4,966,043.5 | 17.52 | 5,836,258.5 | 11.22 | 6,491,122.5 | 4.83 | 6,804,402.5 | 43.42 |
| MnoP | 13,296,947.5 | 1.97 | 13,558,885.0 | 15.55 | 15,667,256.5 | 10.44 | 17,303,212.5 | 3.82 | 17,963,337.5 | 35.09 |
| Intermediate patients | 885,075.5 | 8.94 | 964,191.5 | 17.83 | 1,136,137.5 | 12.02 | 1,272,714.0 | 5.36 | 1,340,875.5 | 51.50 |
| Mentally ill | 18,926,261.5 | 2.97 | 19,489,120.0 | 16.17 | 22,639,652.5 | 10.72 | 25,067,049.0 | 4.16 | 26,108,615.5 | 37.95 |
| Included patients | 19,231,441.0 | 2.89 | 19,786,637.0 | 16.77 | 23,105,266.5 | 11.17 | 25,685,864.0 | 4.22 | 26,770,917.5 | 39.20 |

**Suppl. Tab. 6. Percentage of services billed for specific diagnosis groups and the differences in the proportions between the years or between 2015 and 2019 - differentiated by diagnosis group.**

| Diagnosis group | Percentage 2015 | Difference 2015-2016 in % | Percentage 2016 | Difference 2016-2017 in % | Percentage 2017 | Difference 2017-2018 in % | Percentage 2018 | Difference 2018-2019 in % | Percentage 2019 | Difference 2015-2019 in % |
| --- | --- | --- | --- | --- | --- | --- | --- | --- | --- | --- |
| cMPs | 25.07 | 0.41 | 25.48 | 0.30 | 25.78 | 0.12 | 25.90 | 0.17 | 26.06 | 0.99 |
| MnoP | 70.26 | -0.69 | 69.57 | -0.37 | 69.20 | -0.18 | 69.03 | -0.23 | 68.80 | -1.45 |
| Intermediate patients | 4.68 | 0.27 | 4.95 | 0.07 | 5.02 | 0.06 | 5.08 | 0.06 | 5.14 | 0.46 |

**Suppl. Tab. 7. Percentage of mentally ill patients in the individual diagnosis groups and differences in these proportions between the years or between 2015 and 2019 - separated by two age groups and gender.**

| Age | Gender | Diagnosis group | Percentage 2015 | Difference 2015-2016 in % | Percentage 2016 | Difference 2016-2017 in % | Percentage 2017 | Difference 2017-2018 in % | Percentage 2018 | Difference 2018-2019 in % | Percentage 2019 | Difference 2015-2019 in % |
| --- | --- | --- | --- | --- | --- | --- | --- | --- | --- | --- | --- | --- |
| 18-49  years | Female | cMPs | 18.65 | 0.22 | 18.69 | -0.32 | 18.63 | -1.03 | 18.44 | -0.79 | 18.29 | -1.91 |
|  |  | MnoP | 77.75 | -0.31 | 77.50 | 0.01 | 77.51 | 0.24 | 77.70 | 0.14 | 77.81 | 0.08 |
|  | Male | cMPs | 19.25 | -0.93 | 19.07 | -1.45 | 18.80 | -1.89 | 18.44 | -0.81 | 18.30 | -4.97 |
|  |  | MnoP | 77.88 | 0.08 | 77.94 | 0.36 | 78.22 | 0.48 | 78.60 | 0.18 | 78.74 | 1.10 |
| 50-79 years | Female | cMPs | 39.09 | 1.02 | 39.49 | 1.39 | 40.04 | 1.43 | 40.61 | 1.20 | 41.10 | 5.13 |
|  |  | MnoP | 53.41 | -1.19 | 52.77 | -1.19 | 52.15 | -1.24 | 51.50 | -1.12 | 50.92 | -4.66 |
|  | Male | cMPs | 42.20 | 1.57 | 42.86 | 1.04 | 43.31 | 1.47 | 43.94 | 1.25 | 44.49 | 5.43 |
|  |  | MnoP | 50.76 | -1.66 | 49.92 | -1.16 | 49.34 | -1.56 | 48.57 | -1.38 | 47.90 | -5.64 |

**Suppl. Tab. 8. Absolute frequencies (a. f.) of the included patients as well as percentage change (p.c.) in each year relative to the previous year and percentage change (p. c.) in 2019 relative to 2015 - differentiated by age, sex and diagnosis group of the treated patients.**

| Age | Gender | Diagnosis group | a. f. 2015 | p. c 2015 -2016 | a. f.  2016 | p. c.  2016 -2017 | a. f. 2017 | p. c. 2017 -2018 | a. f. 2018 | p. c. 2018 -2019 | a. f. 2019 | p. c. 2015 -2019 |
| --- | --- | --- | --- | --- | --- | --- | --- | --- | --- | --- | --- | --- |
| 18-19 years | Female | cMPs | 3,673 | 3.29 | 3,794 | 12.84 | 4,281 | 3.01 | 4,410 | 2.88 | 4,537 | 23.52 |
|  |  | MnoP | 24,572 | 7.46 | 26,404 | 14.50 | 30,233 | 10.42 | 33,382 | 4.45 | 34,868 | 41.90 |
|  |  | Intermediate patients | 468 | 10.26 | 516 | 10.08 | 568 | 4.93 | 596 | 3.86 | 619 | 32.26 |
|  |  | Mentally ill | 28,713 | 6.97 | 30,714 | 14.22 | 35,082 | 9.42 | 38,388 | 4.26 | 40,024 | 39.39 |
|  |  | Included patients | 33,436 | 5.77 | 35,364 | 15.87 | 40,977 | 11.09 | 45,523 | 4.47 | 47,560 | 42.24 |
|  | Male | cMPs | 1,449 | 2.83 | 1,490 | 18.59 | 1,767 | 10.75 | 1,957 | 0.97 | 1,976 | 36.37 |
|  |  | MnoP | 9,513 | 8.20 | 10,293 | 28.65 | 13,242 | 9.73 | 14,531 | 0.06 | 14,539 | 52.83 |
|  |  | Intermediate patients | 92 | 27.17 | 117 | 24.79 | 146 | 13.70 | 166 | 3.01 | 171 | 85.87 |
|  |  | Mentally ill | 11,054 | 7.65 | 11,900 | 27.35 | 15,155 | 9.89 | 16,654 | 0.19 | 16,686 | 50.95 |
|  |  | Included patients | 14,690 | 7.00 | 15,719 | 28.48 | 20,196 | 13.62 | 22,947 | 0.90 | 23,153 | 57.61 |
|  | Total | cMPs | 5,122 | 3.16 | 5,284 | 14.46 | 6,048 | 5.27 | 6,367 | 2.29 | 6,513 | 27.16 |
|  |  | MnoP | 34,085 | 7.66 | 36,697 | 18.47 | 43,475 | 10.21 | 47,913 | 3.12 | 49,407 | 44.95 |
|  |  | Intermediate patients | 560 | 13.04 | 633 | 12.80 | 714 | 6.72 | 762 | 3.67 | 790 | 41.07 |
|  |  | Mentally ill | 39,767 | 7.16 | 42,614 | 17.89 | 50,237 | 9.56 | 55,042 | 3.03 | 56,710 | 42.61 |
|  |  | Included patients | 48,126 | 6.14 | 51,083 | 19.75 | 61,173 | 11.93 | 68,470 | 3.28 | 70,713 | 46.93 |
| 20-29 years | Female | cMPs | 23,536 | 1.92 | 23,987 | 11.51 | 26,748 | 5.73 | 28,280 | 2.49 | 28,983 | 23.14 |
|  |  | MnoP | 147,623 | 1.19 | 149,378 | 12.34 | 167,811 | 8.17 | 181,529 | 5.10 | 190,781 | 29.24 |
|  |  | Intermediate patients | 4,205 | 8.04 | 4,543 | 12.08 | 5,092 | 8.74 | 5,537 | 3.76 | 5,745 | 36.62 |
|  |  | Mentally ill | 175,364 | 1.45 | 177,908 | 12.22 | 199,651 | 7.86 | 215,346 | 4.72 | 225,509 | 28.59 |
|  |  | Included patients | 190,471 | 1.15 | 192,659 | 13.99 | 219,613 | 9.11 | 239,629 | 4.98 | 251,568 | 32.08 |

| Age | Gender | Diagnosis group | a. f. 2015 | p. c. 2015 -2016 | a. f.  2016 | p. c.  2016 -2017 | a. f. 2017 | p. c. 2017 -2018 | a. f. 2018 | p. c. 2018 -2019 | a. f. 2019 | p. c. 2015 -2019 |
| --- | --- | --- | --- | --- | --- | --- | --- | --- | --- | --- | --- | --- |
| 20-29 years | Male | cMPs | 9,491 | 4.71 | 9,938 | 15.54 | 11,482 | 9.16 | 12,534 | 7.59 | 13,485 | 42.08 |
|  |  | MnoP | 60,507 | 5.90 | 64,075 | 18.06 | 75,644 | 12.84 | 85,354 | 7.04 | 91,366 | 51.00 |
|  |  | Intermediate patients | 1,083 | 9.33 | 1,184 | 12.92 | 1,337 | 19.15 | 1,593 | 5.52 | 1,681 | 55.22 |
|  |  | Mentally ill | 71,081 | 5.79 | 75,197 | 17.64 | 88,463 | 12.45 | 99,481 | 7.09 | 106,532 | 49.87 |
|  |  | Included patients | 82,189 | 5.24 | 86,497 | 20.21 | 103,974 | 13.80 | 118,320 | 7.65 | 127,377 | 54.98 |
|  | Total | cMPs | 33,027 | 2.72 | 33,925 | 12.69 | 38,230 | 6.76 | 40,814 | 4.05 | 42,468 | 28.59 |
|  |  | MnoP | 208,130 | 2.56 | 213,453 | 14.06 | 243,455 | 9.62 | 266,883 | 5.72 | 282,147 | 35.56 |
|  |  | Intermediate patients | 5,288 | 8.30 | 5,727 | 12.26 | 6,429 | 10.90 | 7,130 | 4.15 | 7,426 | 40.43 |
|  |  | Mentally ill | 246,445 | 2.70 | 253,105 | 13.83 | 288,114 | 9.27 | 314,827 | 5.47 | 332,041 | 34.73 |
|  |  | Included patients | 272,660 | 2.38 | 279,156 | 15.92 | 323,587 | 10.62 | 357,949 | 5.87 | 378,945 | 38.98 |
| 30-39  years | Female | cMPs | 39,655 | 2.57 | 40,674 | 9.19 | 44,410 | 6.67 | 47,373 | 3.78 | 49,163 | 23.98 |
|  |  | MnoP | 189,278 | 1.50 | 192,123 | 9.72 | 210,790 | 6.89 | 225,312 | 3.97 | 234,265 | 23.77 |
|  |  | Intermediate patients | 7,829 | 10.24 | 8,631 | 13.60 | 9,805 | 7.50 | 10,540 | 6.00 | 11,172 | 42.70 |
|  |  | Mentally ill | 236,762 | 1.97 | 241,428 | 9.77 | 265,005 | 6.88 | 283,225 | 4.02 | 294,600 | 24.43 |
|  |  | Included patients | 250,077 | 1.77 | 254,492 | 10.93 | 282,314 | 7.82 | 304,402 | 4.25 | 317,336 | 26.90 |
|  | Male | cMPs | 15,988 | 4.93 | 16,777 | 13.14 | 18,982 | 8.92 | 20,675 | 5.92 | 21,899 | 36.97 |
|  |  | MnoP | 76,732 | 4.50 | 80,185 | 14.66 | 91,939 | 10.19 | 101,311 | 6.45 | 107,847 | 40.55 |
|  |  | Intermediate patients | 2,236 | 12.88 | 2,524 | 23.49 | 3,117 | 10.97 | 3,459 | 8.93 | 3,768 | 68.52 |
|  |  | Mentally ill | 94,956 | 4.77 | 99,486 | 14.63 | 114,038 | 10.00 | 125,445 | 6.43 | 133,514 | 40.61 |
|  |  | Included patients | 104,591 | 4.32 | 109,106 | 16.50 | 127,110 | 11.23 | 141,384 | 6.89 | 151,123 | 44.49 |
|  | Total | cMPs | 55,643 | 3.25 | 57,451 | 10.34 | 63,392 | 7.34 | 68,048 | 4.43 | 71,062 | 27.71 |
|  |  | MnoP | 266,010 | 2.37 | 272,308 | 11.17 | 302,729 | 7.89 | 326,623 | 4.74 | 342,112 | 28.61 |
|  |  | Intermediate patients | 10,065 | 10.83 | 11,155 | 15.84 | 12,922 | 8.33 | 13,999 | 6.72 | 14,940 | 48.44 |
|  |  | Mentally ill | 331,718 | 2.77 | 340,914 | 11.18 | 379,043 | 7.82 | 408,670 | 4.76 | 428,114 | 29.06 |
|  |  | Included patients | 354,668 | 2.52 | 363,598 | 12.60 | 409,424 | 8.88 | 445,786 | 5.09 | 468,459 | 32.08 |

| Age | Gender | Diagnosis group | a. f. 2015 | p. c. 2015 -2016 | a. f.  2016 | p. c.  2016 -2017 | a. f. 2017 | p. c. 2017 -2018 | a. f. 2018 | p. c. 2018 -2019 | a. f. 2019 | p. c. 2015 -2019 |
| --- | --- | --- | --- | --- | --- | --- | --- | --- | --- | --- | --- | --- |
| 40-49  years | Female | cMPs | 66,515 | -1.60 | 65,451 | 7.43 | 70,314 | 2.40 | 72,004 | 1.06 | 72,769 | 9.40 |
|  |  | MnoP | 194,577 | -3.71 | 187,360 | 5.43 | 197,532 | 1.53 | 200,560 | 0.39 | 201,337 | 3.47 |
|  |  | Intermediate patients | 13,276 | 2.26 | 13,576 | 8.41 | 14,718 | 3.28 | 15,201 | 2.61 | 15,597 | 17.48 |
|  |  | Mentally ill | 274,368 | -2.91 | 266,387 | 6.07 | 282,564 | 1.84 | 287,765 | 0.67 | 289,703 | 5.59 |
|  |  | Included patients | 285,752 | -3.10 | 276,885 | 6.72 | 295,485 | 2.50 | 302,885 | 0.82 | 305,382 | 6.87 |
|  | Male | cMPs | 28,506 | -1.27 | 28,143 | 10.24 | 31,024 | 3.46 | 32,096 | 1.46 | 32,564 | 14.24 |
|  |  | MnoP | 77,490 | -2.31 | 75,700 | 8.83 | 82,387 | 3.71 | 85,447 | 2.04 | 87,194 | 12.52 |
|  |  | Intermediate patients | 4,840 | 3.04 | 4,987 | 8.76 | 5,424 | 2.60 | 5,565 | 2.61 | 5,710 | 17.98 |
|  |  | Mentally ill | 110,836 | -1.81 | 108,830 | 9.19 | 118,835 | 3.60 | 123,108 | 1.92 | 125,468 | 13.20 |
|  |  | Included patients | 118,592 | -2.15 | 116,043 | 10.47 | 128,195 | 4.52 | 133,991 | 2.22 | 136,970 | 15.50 |
|  | Total | cMPs | 95,021 | -1.50 | 93,594 | 8.27 | 101,338 | 2.73 | 104,100 | 1.18 | 105,333 | 10.85 |
|  |  | MnoP | 272,067 | -3.31 | 263,060 | 6.41 | 279,919 | 2.17 | 286,007 | 0.88 | 288,531 | 6.05 |
|  |  | Intermediate patients | 18,116 | 2.47 | 18,563 | 8.51 | 20,142 | 3.10 | 20,766 | 2.61 | 21,307 | 17.61 |
|  |  | Mentally ill | 385,204 | -2.59 | 375,217 | 6.98 | 401,399 | 2.36 | 410,873 | 1.05 | 415,171 | 7.78 |
|  |  | Included patients | 404,344 | -2.82 | 392,928 | 7.83 | 423,680 | 3.11 | 436,876 | 1.25 | 442,352 | 9.40 |
| 50-59  years | Female | cMPs | 103,540 | 5.68 | 109,426 | 14.62 | 125,427 | 8.79 | 136,458 | 5.12 | 143,438 | 38.53 |
|  |  | MnoP | 177,188 | 3.92 | 184,141 | 11.99 | 206,222 | 6.35 | 219,309 | 2.77 | 225,373 | 27.19 |
|  |  | Intermediate patients | 20,053 | 8.86 | 21,830 | 14.30 | 24,951 | 9.51 | 27,323 | 5.57 | 28,844 | 43.84 |
|  |  | mentally ill | 300,781 | 4.86 | 315,397 | 13.06 | 356,600 | 7.43 | 383,090 | 3.80 | 397,655 | 32.21 |
|  |  | included patients | 310,372 | 4.58 | 324,596 | 13.68 | 368,995 | 7.91 | 398,189 | 3.74 | 413,066 | 33.09 |
|  | Male | cMPs | 45,592 | 7.39 | 48,960 | 15.96 | 56,775 | 10.38 | 62,669 | 5.46 | 66,091 | 44.96 |
|  |  | MnoP | 67,626 | 4.63 | 70,756 | 13.71 | 80,458 | 8.15 | 87,018 | 3.71 | 90,249 | 33.45 |
|  |  | Intermediate patients | 7,922 | 7.50 | 8,516 | 17.53 | 10,009 | 12.57 | 11,267 | 5.05 | 11,836 | 49.41 |
|  |  | Mentally ill | 121,140 | 5.85 | 128,232 | 14.82 | 147,242 | 9.31 | 160,954 | 4.49 | 168,176 | 38.83 |
|  |  | Included patients | 127,290 | 5.66 | 134,492 | 15.61 | 155,487 | 9.87 | 170,839 | 4.53 | 178,571 | 40.29 |

| Age | Gender | Diagnosis group | a. f. 2015 | p. c. 2015 -2016 | a. f.  2016 | p. c.  2016 -2017 | a. f. 2017 | p. c. 2017 -2018 | a. f. 2018 | p. c. 2018 -2019 | a. f. 2019 | p. c. 2015 -2019 |
| --- | --- | --- | --- | --- | --- | --- | --- | --- | --- | --- | --- | --- |
| 50-59 years | Total | cMPs | 149,132 | 6.21 | 158,386 | 15.04 | 182,202 | 9.29 | 199,127 | 5.22 | 209,529 | 40.50 |
|  |  | MnoP | 244,814 | 4.12 | 254,897 | 12.47 | 286,680 | 6.85 | 306,327 | 3.03 | 315,622 | 28.92 |
|  |  | Intermediate patients | 27,975 | 8.48 | 30,346 | 15.20 | 34,960 | 10.38 | 38,590 | 5.42 | 40,680 | 45.42 |
|  |  | Mentally ill | 421,921 | 5.15 | 443,629 | 13.57 | 503,842 | 7.98 | 544,044 | 4.00 | 565,831 | 34.11 |
|  |  | Included patients | 437,662 | 4.90 | 459,088 | 14.24 | 524,482 | 8.49 | 569,028 | 3.97 | 591,637 | 35.18 |
| 60-69  years | Female | cMPs | 50,958 | 9.94 | 56,025 | 20.41 | 67,458 | 15.28 | 77,766 | 10.69 | 86,079 | 68.92 |
|  |  | MnoP | 49,331 | 8.35 | 53,449 | 19.73 | 63,996 | 13.67 | 72,745 | 10.77 | 80,580 | 63.35 |
|  |  | Intermediate patients | 9,393 | 13.36 | 10,648 | 20.52 | 12,833 | 12.84 | 14,481 | 10.84 | 16,051 | 70.88 |
|  |  | Mentally ill | 109,682 | 9.52 | 120,122 | 20.12 | 144,287 | 14.35 | 164,992 | 10.74 | 182,710 | 66.58 |
|  |  | Included patients | 113,164 | 9.18 | 123,558 | 20.64 | 149,065 | 14.88 | 171,244 | 10.59 | 189,379 | 67.35 |
|  | Male | cMPs | 20,653 | 10.94 | 22,912 | 22.30 | 28,021 | 18.69 | 33,257 | 13.66 | 37,799 | 83.02 |
|  |  | MnoP | 16,703 | 6.16 | 17,732 | 22.32 | 21,689 | 16.27 | 25,217 | 12.15 | 28,280 | 69.31 |
|  |  | Intermediate patients | 3,248 | 14.04 | 3,704 | 23.43 | 4,572 | 17.24 | 5,360 | 17.29 | 6,287 | 93.57 |
|  |  | Mentally ill | 40,604 | 9.22 | 44,348 | 22.40 | 54,282 | 17.60 | 63,834 | 13.37 | 72,366 | 78.22 |
|  |  | Included patients | 42,704 | 8.86 | 46,488 | 22.84 | 57,107 | 18.34 | 67,579 | 13.12 | 76,445 | 79.01 |
|  | Total | cMPs | 71,611 | 10.23 | 78,937 | 20.96 | 95,479 | 16.28 | 111,023 | 11.58 | 123,878 | 72.99 |
|  |  | MnoP | 66,034 | 7.79 | 71,181 | 20.38 | 85,685 | 14.33 | 97,962 | 11.12 | 108,860 | 64.85 |
|  |  | Intermediate patients | 12,641 | 13.54 | 14,352 | 21.27 | 17,405 | 14.00 | 19,841 | 12.59 | 22,338 | 76.71 |
|  |  | Mentally ill | 150,286 | 9.44 | 164,470 | 20.73 | 198,569 | 15.24 | 228,826 | 11.47 | 255,076 | 69.73 |
|  |  | Included patients | 155,868 | 9.10 | 170,046 | 21.24 | 206,172 | 15.84 | 238,823 | 11.31 | 265,824 | 70.54 |
| 70-79  years | Female | cMPs | 18,024 | 6.32 | 19,164 | 18.51 | 22,711 | 11.31 | 25,280 | 8.29 | 27,375 | 51.88 |
|  |  | MnoP | 9,210 | -0.89 | 9,128 | 15.94 | 10,583 | 10.30 | 11,673 | 5.75 | 12,344 | 34.03 |
|  |  | Intermediate patients | 3,648 | 1.26 | 3,694 | 16.78 | 4,314 | 9.78 | 4,736 | 5.70 | 5,006 | 37.23 |
|  |  | Mentally ill | 30,882 | 3.57 | 31,986 | 17.58 | 37,608 | 10.85 | 41,689 | 7.28 | 44,725 | 44.83 |
|  |  | Included patients | 32,431 | 3.27 | 33,490 | 18.08 | 39,546 | 11.37 | 44,041 | 7.13 | 47,179 | 45.48 |

| Age | Gender | Diagnosis group | a. f. 2015 | p. c. 2015 -2016 | a. f.  2016 | p. c.  2016 -2017 | a. f. 2017 | p. c. 2017 -2018 | a. f. 2018 | p. c. 2018 -2019 | a. f. 2019 | p. c. 2015 -2019 |
| --- | --- | --- | --- | --- | --- | --- | --- | --- | --- | --- | --- | --- |
| 70-79 years | Male | cMPs | 5,487 | 5.08 | 5,766 | 18.70 | 6,844 | 15.87 | 7,930 | 9.86 | 8,712 | 58.78 |
|  |  | MnoP | 1,952 | -1.28 | 1,927 | 17.13 | 2,257 | 12.98 | 2,550 | 5.33 | 2,686 | 37.60 |
|  |  | Intermediate patients | 796 | 8.04 | 860 | 14.19 | 982 | 8.66 | 1,067 | 6.75 | 1,139 | 43.09 |
|  |  | Mentally ill | 8,235 | 3.86 | 8,553 | 17.89 | 10,083 | 14.52 | 11,547 | 8.57 | 12,537 | 52.24 |
|  |  | Included patients | 9,018 | 3.16 | 9,303 | 18.70 | 11,043 | 14.89 | 12,687 | 8.14 | 13,720 | 52.14 |
|  | Total | cMPs | 23,511 | 6.04 | 24,930 | 18.55 | 29,555 | 12.37 | 33,210 | 8.66 | 36,087 | 53.49 |
|  |  | MnoP | 11,162 | -0.96 | 11,055 | 16.15 | 12,840 | 10.77 | 14,223 | 5.67 | 15,030 | 34.65 |
|  |  | Intermediate patients | 4,444 | 2.48 | 4,554 | 16.29 | 5,296 | 9.57 | 5,803 | 5.89 | 6,145 | 38.28 |
|  |  | Mentally ill | 39,117 | 3.64 | 40,539 | 17.64 | 47,691 | 11.63 | 53,236 | 7.56 | 57,262 | 46.39 |
|  |  | Included patients | 41,449 | 3.24 | 42,793 | 18.22 | 50,589 | 12.14 | 56,728 | 7.35 | 60,899 | 46.93 |
| Total | Female | cMPs | 305,901 | 4.13 | 318,521 | 13.45 | 361,349 | 8.36 | 391,571 | 5.31 | 412,344 | 34.80 |
|  |  | MnoP | 791,779 | 1.29 | 801,983 | 10.62 | 887,167 | 6.46 | 944,510 | 3.71 | 979,548 | 23.71 |
|  |  | Intermediate patients | 58,872 | 7.76 | 63,438 | 13.94 | 72,281 | 8.48 | 78,414 | 5.89 | 83,034 | 41.04 |
|  |  | Mentally ill | 1,156,552 | 2.37 | 1,183,942 | 11.56 | 1,320,797 | 7.09 | 1,414,495 | 4.27 | 1,474,926 | 27.53 |
|  |  | Included patients | 1,215,703 | 2.08 | 1,241,044 | 12.49 | 1,395,995 | 7.87 | 1,505,913 | 4.35 | 1,571,470 | 29.26 |
|  | Male | cMPs | 127,166 | 5.36 | 133,986 | 15.61 | 154,895 | 10.47 | 171,118 | 6.67 | 182,526 | 43.53 |
|  |  | MnoP | 310,523 | 3.27 | 320,668 | 14.64 | 367,616 | 9.20 | 401,428 | 5.16 | 422,161 | 35.95 |
|  |  | intermediate patients | 20,217 | 8.29 | 21,892 | 16.88 | 25,587 | 11.29 | 28,477 | 7.43 | 30,592 | 51.32 |
|  |  | mentally ill | 457,906 | 4.07 | 476,546 | 15.01 | 548,098 | 9.66 | 601,023 | 5.70 | 635,279 | 38.74 |
|  |  | included patients | 499,074 | 3.72 | 517,648 | 16.51 | 603,112 | 10.72 | 667,747 | 5.93 | 707,359 | 41.73 |
|  | Total | cMPs | 433,067 | 4.49 | 452,507 | 14.09 | 516,244 | 9.00 | 562,689 | 5.72 | 594,870 | 37.36 |
|  |  | MnoP | 1,102,302 | 1.85 | 1,122,651 | 11.77 | 1,254,783 | 7.26 | 1,345,938 | 4.14 | 1,401,709 | 27.16 |
|  |  | Intermediate patients | 79,089 | 7.89 | 85,330 | 14.69 | 97,868 | 9.22 | 106,891 | 6.30 | 113,626 | 43.67 |
|  |  | Mentally ill | 1,614,458 | 2.85 | 1,660,488 | 12.55 | 1,868,895 | 7.85 | 2,015,518 | 4.70 | 2,110,205 | 30.71 |
|  |  | Included patients | 1,714,777 | 2.56 | 1,758,692 | 13.67 | 1,999,107 | 8.73 | 2,173,660 | 4.84 | 2,278,829 | 32.89 |

**Suppl. Tab. 9. Percentage of billed services for diagnosis groups - differentiated by two age cohorts and gender.**

| Age | Gender | Diagnosis group | Percentage 2015 | Difference 2015 -2016 in % | Percentage 2016 | Difference 2016 -2017 in % | Percentage 2017 | Difference 2017 -2018 in % | Percentage 2018 | Difference 2018 -2019 in % | P  Percentage 2019 | Difference 2015 -2019 in % |
| --- | --- | --- | --- | --- | --- | --- | --- | --- | --- | --- | --- | --- |
| 18-49  years | Female | cMPs | 17.97 | 0.01 | 17.97 | -0.25 | 17.93 | -1.36 | 17.69 | -1.17 | 17.48 | -2.75 |
|  |  | MnoP | 78.51 | -0.29 | 78.29 | 0.08 | 78.35 | 0.27 | 78.56 | 0.26 | 78.76 | 0.32 |
|  | Male | cMPs | 18.48 | -0.73 | 18.35 | -1.43 | 18.09 | -2.27 | 17.68 | -1.45 | 17.42 | -5.76 |
|  |  | MnoP | 78.74 | 0.08 | 78.80 | 0.36 | 79.09 | 0.58 | 79.55 | 0.32 | 79.80 | 1.34 |
| 50-79  years | Female | cMPs | 37.34 | 1.27 | 37.81 | 1.53 | 38.39 | 0.96 | 38.76 | 1.56 | 39.37 | 5.42 |
|  |  | MnoP | 55.28 | -1.38 | 54.52 | -1.26 | 53.83 | -0.78 | 53.41 | -1.43 | 52.65 | -4.76 |
|  | Male | cMPs | 40.20 | 1.77 | 40.91 | 0.73 | 41.21 | 1.33 | 41.76 | 1.41 | 42.35 | 5.33 |
|  |  | MnoP | 52.97 | -2.01 | 51.91 | -0.98 | 51.40 | -1.34 | 50.71 | -1.20 | 50.10 | -5.42 |

**Suppl. Tab. 10. Absolute frequencies of outpatient psychotherapeutic services billed in the years 2015 to 2019, as well as percentage change in each year relative to the previous year and percentage change in 2019 relative to 2015 - differentiated according to inclusion and exclusion criteria.**

| Recipient of service | Absolute frequencies 2015 | Change  2015-2016  in % | Absolute frequencies 2016 | Change  2016-2015 in % | Absolute frequencies 2017 | Change  2017-2018 in % | Absolute frequencies 2018 | Change  2018-2019 in % | Absolute frequencies 2019 | Change  2015-2019 in % |
| --- | --- | --- | --- | --- | --- | --- | --- | --- | --- | --- |
| <18 years | 3,029,981 | 2.60 | 3,108,660.0 | 22.77 | 3,816,352.0 | 16.05 | 4,428,879.0 | 6.20 | 4,703,533.5 | 55.23 |
| >79 years | 52,023 | 16.71 | 60,714.5 | 41.41 | 85,858.5 | 34.36 | 115,360.5 | 16.55 | 134,455.0 | 158.45 |
| F00-09 | 374,830 | 2.92 | 385,764.5 | 19.10 | 459,426.5 | 11.02 | 510,061.5 | 3.07 | 525,703.5 | 40.25 |
| F70-79 | 131,502 | 1.91 | 134,013.5 | 22.88 | 164,669.5 | 13.16 | 186,347.5 | 5.97 | 197,480.0 | 50.17 |
| excluded* | 3,504,131 | 3.00 | 3,609,429.5 | 22.49 | 4,421,328.5 | 15.70 | 5,115,362.5 | 6.21 | 5,433,044.5 | 55.05 |
| included | 19,231,441 | 2.89 | 19,786,637.0 | 16.77 | 23,105,266.5 | 11.17 | 25,685,864.0 | 4.22 | 26,770,917.5 | 39.20 |
| mentally ill | 18,926,262 | 2.97 | 19,489,120.0 | 16.17 | 22,639,652.5 | 10.72 | 25,067,049.0 | 4.16 | 26,108,615.5 | 37.95 |
| KBV-record | 22,735,572 | 2.91 | 23,396,066.5 | 17.65 | 27,526,595.0 | 11.90 | 30,801,226.5 | 4.55 | 32,203,962.0 | 41.65 |

* Subsumes all services which were billed for all excluded patients (i.e. <18 years , >79 years, F00-09, F70-79)
